# Whole Genome Characterization of a Few EMS-Induced Mutants of Upland Rice Variety Nagina 22 Reveals a Staggeringly High Frequency of SNPs Which Show High Phenotypic Plasticity Towards the Wild-Type

**DOI:** 10.3389/fpls.2018.01179

**Published:** 2018-09-04

**Authors:** Amitha M. V. Sevanthi, Prashant Kandwal, Prashant B. Kale, Chandra Prakash, M. K. Ramkumar, Neera Yadav, Ajay K. Mahato, V. Sureshkumar, Motilal Behera, Rupesh K. Deshmukh, P. Jeyaparakash, Meera K. Kar, S. Manonmani, Raveendran Muthurajan, K. S. Gopala, Sarla Neelamraju, M. S. Sheshshayee, P. Swain, Ashok K. Singh, N. K. Singh, Trilochan Mohapatra, R. P. Sharma

**Affiliations:** ^1^ICAR-National Research Centre on Plant Biotechnology, New Delhi, India; ^2^ICAR-National Rice Research Institute, Cuttack, India; ^3^Laval University, Quebec, QC, Canada; ^4^Tamil Nadu Agricultural University, Coimbatore, India; ^5^ICAR-Indian Agricultural Research Institute, New Delhi, India; ^6^ICAR-Indian Institute of Rice Research, Hyderabad, India; ^7^University of Agricultural Sciences, Bengaluru, India; ^8^Indian Council of Agriculture Research, New Delhi, India

**Keywords:** EMS mutants, rice, Nagina 22, mutant resource, ionomics, mutation frequency

## Abstract

The Indian initiative, in creating mutant resources for the functional genomics in rice, has been instrumental in the development of 87,000 ethylmethanesulfonate (EMS)-induced mutants, of which 7,000 are in advanced generations. The mutants have been created in the background of Nagina 22, a popular drought- and heat-tolerant upland cultivar. As it is a pregreen revolution cultivar, as many as 573 dwarf mutants identified from this resource could be useful as an alternate source of dwarfing. A total of 541 mutants, including the macromutants and the trait-specific ones, obtained after appropriate screening, are being maintained in the mutant garden. Here, we report on the detailed characterizations of the 541 mutants based on the distinctness, uniformity, and stability (DUS) descriptors at two different locations. About 90% of the mutants were found to be similar to the wild type (WT) with high similarity index (>0.6) at both the locations. All 541 mutants were characterized for chlorophyll and epicuticular wax contents, while a subset of 84 mutants were characterized for their ionomes, namely, phosphorous, silicon, and chloride contents. Genotyping of these mutants with 54 genomewide simple sequence repeat (SSR) markers revealed 93% of the mutants to be either completely identical to WT or nearly identical with just one polymorphic locus. Whole genome resequencing (WGS) of four mutants, which have minimal differences in the SSR fingerprint pattern and DUS characters from the WT, revealed a staggeringly high number of single nucleotide polymorphisms (SNPs) on an average (16,453 per mutant) in the genic sequences. Of these, nearly 50% of the SNPs led to non-synonymous codons, while 30% resulted in synonymous codons. The number of insertions and deletions (InDels) varied from 898 to 2,595, with more than 80% of them being 1–2 bp long. Such a high number of SNPs could pose a serious challenge in identifying gene(s) governing the mutant phenotype by next generation sequencing-based mapping approaches such as Mutmap. From the WGS data of the WT and the mutants, we developed a genic resource of the WT with a novel analysis pipeline. The entire information about this resource along with the panicle architecture of the 493 mutants is made available in a mutant database EMS*garde*N22 (http://14.139.229.201/EMSgardeN22).

## Introduction

Rice (*Oryza sativa* L.) is a genomic model crop species for monocots, and is the main staple food for more than 50% of the world’s population (Gross and Zhao, 2014). Structural genomics in rice is well established with multifarious resources (IRGSP, 2005; 3000 rice genome project; Kumar et al., 2015; Singh et al., 2016; Mansueto et al., 2017; Sandhu et al., 2017), while only 2,000 genes have been functionally characterized in rice, indicating that progress in functional genomics is much more laborious and time and resource consuming than initially envisaged. The functionally characterized genes were either identified by traditional genetic- and map-based cloning approaches (Ashikari and Matsuoka, 2006; Miura et al., 2011; Yamamoto et al., 2012; Liu et al., 2015; Neelam et al., 2016) or from mutant resources. As of now, nearly 22 independent rice mutant resources in more than 10 diverse genetic backgrounds, including Nipponbare, Dong Jin, Zhonghua 11, Nagina 22, IR 64, Kasalath, Kitakke, and Hwayoung with a wide range of mutant lines (6,000–500,000) and covering 68% of the genic region, have been created (**Supplementary Table S1**; Krishnan et al., 2009; Wei et al., 2013; Mohapatra et al., 2014; Li et al., 2017). Further, even after a decade of establishing high-quality whole genome sequence resources in rice with 66,638 genes, nearly 28% of the genes, namely 2,421 hypothetical proteins, 198 conserved hypothetical genes, and 16,209 expressed proteins, are yet to be annotated. Hence, there is a need for more concerted efforts to strengthen the functional genomics in rice.

In countries where field evaluations of transgenic plants are under regulatory regime, physical and chemical mutagens are a better choice to create mutant resources (Mohapatra et al., 2014; Amitha Mithra et al., 2016). Among the available physical/chemical mutagenic agents, ethylmethanesulfonate (EMS) has an advantage as it is simple, cheap, and generates a high density of random irreversible point mutations uniformly distributed in the genome (Kim et al., 2004). The EMS alkylates the guanine nucleotide, resulting in mainly G/C to T/A transitions and, at low frequency, A/T to G/C transitions through 3-ethyladenine pairing errors (Kim et al., 2004), inducing 2 to 10 mutations/Mb of diploid DNA and, hence, is considered as an excellent resource for targeting induced local lesions in genomes (TILLING; Till et al., 2007). However, integrating forward and reverse genetics is more prudent/useful in such a resource than employing a reverse genetic technique like TILLING alone, when the sequencing costs have steadily become cheaper over the past decade (Van Dijk et al., 2014). Further, with the advancement in sequencing technology, it has also become possible to index mutants generated by physical mutagens such as fast neutrons, as in the case of T-DNA insertion mutants (Li et al., 2017). To enable effective utilization of physical and chemical mutant resources in functional genomics, high-quality genome sequencing information of the wild type (WT) genotype is essential.

Nagina 22 (N22) is a pregreen revolution, tall, and poor nitrogen and phosphorous (P)-assimilating upland rice cultivar with a very high degree of tolerance to heat and drought and a high CO_2_ compensation point from which useful loss and gain of function mutants can be identified (Poli et al., 2013; Mohapatra et al., 2014; Pradhan et al., 2016). We earlier reported on the generation and morphological characterization of 370 EMS mutants in the background of Nagina 22 (Mohapatra et al., 2014; Amitha Mithra et al., 2016). From this resource, a herbicide (Imazethapyr)-tolerant mutant has recently been identified, characterized, and is being used in breeding herbicide-tolerant varieties (Shoba et al., 2017). Further, mutants with gain and loss of function for drought tolerance, heat tolerance, performance under low P, tillering, and plant height, grain size, and stay-green trait (SGT) have been also been identified (Poli et al., 2013, 2017; Kulkarni et al., 2014; Panigrahy et al., 2014; Lima et al., 2015; Talla et al., 2016). In this article, we report on the advances made in the N22 EMS mutant resources in terms of resource generation, characterization of 541 mutants maintained in the mutant garden at two different locations, development of the genomic resource of the WT, and establishment of a mutant database.

## Materials and Methods

### Plant Material, Mutagenesis, and Maintenance of Rice Mutants

From the earlier efforts on the EMS mutagenesis of Nagina 22 (N22), 22,292 M_2_ mutants were generated in 2008 (**Supplementary Figure S1**), of which 381 stable macromutants were maintained at the mutant garden of ICAR-National Research Centre on Plant Biotechnology (ICAR-NRCPB), New Delhi, and were described for the morphological variations (Mohapatra et al., 2014). Some of the useful mutants identified from this resource and utilized in research and breeding applications have been briefly described elsewhere (Mohapatra et al., 2014). To augment the resource, the M_1_ plants obtained from 1 Kg of N22 seeds treated with EMS (∼0.8% or 65 mM solution) were raised at the Department of Rice, Centre for Plant Breeding and Genetics, Tamil Nadu Agricultural University (TNAU), Coimbatore (11.0152°N, 76.9326°E) to generate a large M_2_ population of about 87,000 plants in 2014 (Amitha Mithra et al., 2016). They were subsequently raised as M_2_ bulks at four different locations, namely, ICAR-Indian Agricultural Research Institute (ICAR-IARI), New Delhi (28.6402°N, 77.1738°E), ICAR-National Rice Research Institute (ICAR-NRRI), Cuttack (20.4539°N, 85.9349°E), VC farm, Mandya, University of Agricultural Sciences (12.5198°N, 76.8673°E), and TNAU, Coimbatore. Currently, 541 advanced generation and stable macromutants are maintained at the mutant garden grown at ICAR-NRCPB, while 87,000 M_2_ mutants are maintained at the seed storage facility at TNAU and ICAR-NRCPB (**Supplementary Figure S2**).

### Raising the Macromutants

Five hundred and forty one EMS mutants of N22 maintained for nearly 8 generations from *Kharif* 2009 to *Kharif* 2016 at the ICAR-NRCPB (ICAR-IARI farm) were raised in 2 × 1.5 m long rows with 20 × 20 cm spacing along with the WT, N22 at two different locations, research farms at ICAR-NRRI, Cuttack and ICAR-NRCPB, New Delhi in *Kharif* 2017. Recommended agronomic practices were followed to raise a healthy transplanted crop.

### Characterization of Agro-Morphological Traits

A set of 44 distinctness, uniformity, and stability (DUS) traits were recorded as per standard descriptors (Shobha Rani et al., 2004) by recording morphological observations at three different growth stages of the crop, namely, vegetative, flowering, and harvest at ICAR-NRRI, Cuttack (NC), while only 15 of these traits could be observed as the crop lodged owing to heavy rains during early grain filling at ICAR-NRCPB, New Delhi (NN). Data on important quantitative characters like plant height (PH; cm), number of productive tillers (NPT), flag leaf length (FLL; cm), and panicle length (PL; cm) were recorded at ICAR-NRCPB for two consecutive years namely *Kharif* 2016 and 2017 (NN16 and NN17). Furthermore, data on days to 50% flowering (DFF) and grain parameters, namely grain length (GL; mm), grain width (GW; mm), and 100 seed weight (SW; g), were additionally recorded during NN17. Grain parameters data were measured according to Shanmugavadivel et al. (2013). Data on five plants from each mutant line were recorded and averaged to obtain the mean performance of the mutants.

### Characterization of Physiological and Biochemical Traits

From each mutant line, the fifth leaf (from top) from three independent plants were sampled at active tillering stage (ICAR-NRCPB mutant garden) and used for the estimation of chlorophyll and epicuticular wax content. Each sample was measured in three biological as well as three technical replicates. To enable high-throughput analysis, both the experiments were carried out in a 96-deep-well plate of 2 ml volume. In each plate, a blank well (water) and N22 samples were maintained as negative and positive control, respectively. For measurement of chlorophyll content, 5 mg of leaf sample was measured and placed in each well to which 1 ml of dimethyl sulfoxide was added separately, and the entire plate was incubated for 24 h at room temperature (RT). Then, an aliquot of 200 μl was transferred to a fresh microtiter plate and the absorbance was measured at 645 and 663 nm using a microplate reader and SkanIt Software 2.4.5 RE (Varioskan Flash, Thermo Scientific, Waltham, MA, United States). Chlorophyll a, chlorophyll b, and total chlorophyll content were calculated according to Arnon’s (1949) equation and Hiscox and Israelstam (1979). For estimation of epicuticular wax content, 100 mg of leaf sample was placed in a well to which 1.5 ml of chloroform was added and incubated for 15 s at RT. Chloroform extract was immediately removed and the digested sample was collected in a fresh 96-well plate and incubated at 90°C until complete evaporation of chloroform. To this, 500 μl of acidic potassium dichromate (K_2_Cr_2_O_7_) was added and incubated for 30 min at 90°C. To this, 1.0 ml of deionized water was added after cooling to RT, allowed for color development, and the absorbance of the 200 μl aliquot was measured at 590 nm using a microplate reader and SkanIt Software 2.4.5 RE (Varioskan Flash, Thermo Scientific, Waltham, MA, United States). To estimate the wax content of the sample, a standard graph was prepared using carnauba wax (Sigma Aldrich) at different concentrations ranging from 1.0 to 10 mg (González and Ayerbe, 2010).

A random subset of 84 mutants and N22 were selected for estimation of the elemental analysis using portable X-ray fluorescence (XRF). The XRF works on the principle of excitation of inner orbit electrons by an X-ray radiation source. As the excited electrons relax to the ground state, they fluoresce, thereby, ejecting photons of energy and wavelength characteristic of the atoms present (Reidinger et al., 2012). From the *Kharif* 2016 harvest, six to eight individual plant harvests from each mutant were sampled. After anthesis at *Kharif* 2016, these plants neither had any nutritional supplement nor pesticide spray. The husks was separated from seeds and dried in a hot air-oven and further ground to a fine and uniform powder using a steel ball mill homogenizer. Approximately 300 mg husk powder was compacted into uniform sized pellets by applying uniformly 10 tons pressure for 5 s using a manually operated hydraulic press. The compressed pellets with a diameter of 13 mm and thickness of 5 mm were used for XRF spectrometry analysis. A portable XRF machine with a 50 kV X-ray tube and light filter was used to quantify P, Si, and Cl elements in pellets. To convert XRF readings into element percent dry weight, standard curves prepared with peach leaf tissue pellets spiked with a known concentration of elements were used.

### DNA Extraction and Genotyping With Genomewide Microsatellite Markers

From each mutant and N22 grown during *Kharif* 2016, fresh, young, and green leaf samples from five plants were collected, pooled, and stored at -80°C. The DNA was extracted by standard cetyl trimethylammonium bromide (CTAB) procedure (Doyle, 1991). A total of 60 genomewide markers comprising 52 fluorescently labeled simple sequence repeats (SSRs) (labeled with VIC, FAM, NED, and PET) and 8 HvSSRs uniformly distributed across the genome were chosen for fingerprinting the mutants (**Supplementary Figure S3**). The primer details are given in **Supplementary Table S2**. A total PCR reaction volume of 10 μl containing 50 ng of template genomic DNA, 2.0 pmol each of forward and reverse primer, 1 μl of 10X polymerase chain reaction (PCR) buffer, 200 μM each of dNTPs, and 0.5 U of *Taq* DNA polymerase were used for PCR amplification. Four PCR products, each representing one of the four dyes, was mixed in such a way that signal strengths would be equal (Tiwari et al., 2015). One μl of pooled microsatellite sample was mixed with 8.8 μl of Hi–Di formamide and 0.2 μl of an internal sized standard GeneScan^TM^ 500 ROX^TM^ (Applied Biosystems, Foster City, CA, United States). The samples were denatured at 95°C for 5 min, and the amplicons were resolved using the capillary DNA analyzer (3730xl, ABI, CA, United States). Raw results were analyzed with GeneMapper 4.1 software. Allele binning and allele calling were carried out as per the manufacturer’s instructions. The HvSSR PCR products were resolved on 2% agarose (Lonza, Rockland, ME, United States) gels.

### Whole Genome Sequencing of Nagina 22 and Mutants

The DNA was extracted from fresh, young, and healthy leaf samples of N22 and four mutants identified for different traits (drought tolerance, grain chalkiness, and SGT) using the CTAB method. The purified DNA quality was ascertained by Bioanalyzer (Agilent, Santa Clara, CA, United States), and library construction was done using TrueSeq DNA PCR-Free LT kit (Illumina, Singapore). All the five libraries were then individually sequenced by Illumina paired end sequencing technology in HiSeq1000 installed at ICAR-NRCPB. The average library size was 350 bp. The N22 DNA was further sequenced using MiSeq platform (Illumina, Singapore) through AgriGenome Labs Pvt. Ltd., India.

### Mutant Database: Design and Construction

The EMS*garde*N22, a database for the rice EMS mutants of Nagina 22, was constructed using XAMPP (Apache, MariaDB, PHP, and Perl) server. The MySQL was used to design the backend of the database, while HTML5 and CSS3 were used for constructing the frontend. User framework was enabled by Jquery and Javascript. PHP5 was used to connect users and server to access queries. The architecture of the database is given in **Supplementary Figure S4**. The database is hosted in the server environment, FUJITSU PRIMERGY-RX600S6 and Windows operating system. The database has search options for retrieving the passport information of the selected mutant, SSR data, DUS data, and major morphological traits. For each morphological/biochemical/other traits, two search options namely high and low are available. High mutants refer to those mutants with trait value more than “N22+1 SD,” while low mutants refer to those with less than “N22-1 SD.” There is a separate tab for accessing the sequence of all the 66,638 rice genes of Nagina 22. Further, images of panicle architecture of 431 mutants are also available. The database can be accessed at http://14.139.229.201/EMSgardeN22.

### Data Analysis

We used R (R Core Team, 2017) package psych (Revelle, 2017) and Microsoft Excel for statistical analysis and graphical representation of the data. Similarity index for mutants based on DUS data was calculated by comparing mutants’ data with N22 (WT) data. A score of “1” was given to a mutant for a particular trait, if mutant and N22 behaved similarly for the trait; otherwise “0” score was assigned, when trait value of the mutant was different from that of WT. Thus, each mutant had a similarity score assigned for all the traits and each trait also had a similarity score across all the 541 mutants. Then, the similarity index was calculated for each trait by calculating the average similarity scores across all the mutants. Following the same reasoning, similarity index was calculated for each mutant, by calculating average similarity scores across a trait. Similarly, using SSRmarker data, marker similarity index for mutants and markers performance index for each marker were calculated by comparing allele size of mutants and N22. A score of 1 and 0 was given based on similarity or dissimilarity, respectively, by comparing the allele size (PCR amplicon size) of mutants with N22.

To make a reference gene set of Nagina 22, reference gene assembly was performed using high-quality Illumina sequence reads from N22 against the Rice genome MSU v.7 (Rice Genome Annotation Project). Software CLC Genomic Workbench (v.7.0.3 CLCbio, Aarhus, Denmark) was used for reference-guided mapping and assembly of high-quality reads, and consensus was called with coverage threshold = 10 and Inset ‘N’ for low coverage region. We refer to this as the first round of assembly. Further, four more rounds of reference-guided mapping assembly using the whole genome sequence data of four mutants were performed sequentially, to see the effect of adding each mutant sequence data on polishing of N22 gene mapping and assembly. Thus, the final round means the fifth round of assembly. Since there is a possibility of mutant-specific bases (single nucleotide polymorphisms; SNPs) overshadowing the N22 sequences, finally, in the sixth round, the N22 reads were again mapped and all SNPs obtained between the fifth round of assembly and the N22 sequences, were replaced with N22 specific bases (**Supplementary Figure S5**). The output obtained after this step was referred to as N22 genic sequences and used in all further studies. To identify the SNPs in each mutants with respect to N22 genic sequences, read mapping workflow was performed using BWA and SAM tools with each mutant, and SNP calling was performed with software VarScan version 2.4.1 (Koboldt et al., 2012) on customized parameters (number of minimum reads = 2; minimum coverage = 10; minimum average quality = 25). To identify the high-quality SNPs, only those SNPs that mapped uniquely to respective reference gene positions in the newly created N22 reference gene set and where the all reads are supporting the variant base with minimum read coverage > = 10 were considered. The variant calling data along with the reference (N22) nucleotide data were imported to MS-Excel and using the option, ‘Pivot Table,’ in which each type of base change with respect to the N22 data (A to G; A to T; and so on) and their frequencies were listed as rows (mutation type) and values respectively, which were used to calculate the overall frequency of transitions and transversions.

## Results

### Variations in the Mutants for Major Agro-Morphological Traits

**FIGURE 1:**
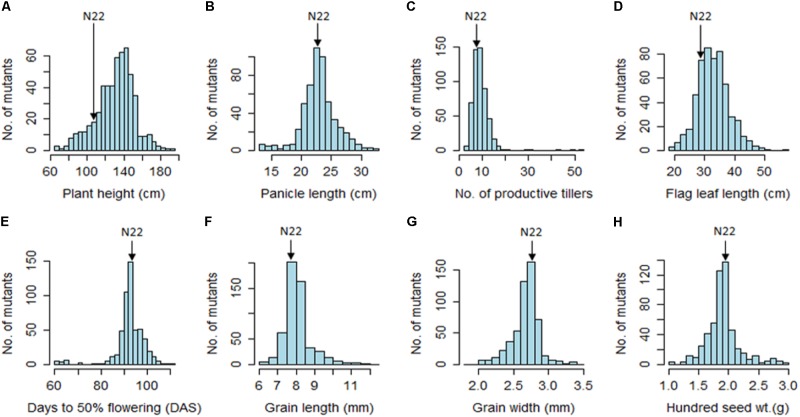
Frequency distribution of 541 rice EMS mutants of Nagina 22 for major morphological traits. **(A)** Plant height; **(B)** panicle length; **(C)** number of productive tillers; **(D)** flag leaf length; **(E)** days to 50% flowering; **(F)** grain length; **(G)** grain width; **(H)** hundred seed weight. Unit of the traits measured are indicated in the parenthesis in the figure.

The EMS mutants of N22 in the mutant garden had shown a huge range of variation for all eight quantitative traits measured, namely, PH (66–190 cm), DFF (61–112 days), NPT (3–53), FLL (19–56.3 cm), PL (13.6–32.6 cm), GL (6–12.3 mm), GW (1.9–3.4 mm), and 100 SW (1.1–3.3 g) (**Figure 1** and **Table 1**). We calculated coefficient of variation (CV) of the mutants using the mean of the mutant garden (CV_MG_) and the mean of the WT N22 (CV_N22_) so as to know the effective variation available. The NPT showed maximum variation (0.51 and 0.61 with respect to CV_MG_ and CV_N22_), while DFF and GW showed the least CV (0.08 CV_MG_ and 0.08CV_N22_) followed by GL (0.10 and 0.11 with respect to CV_MG_ and CV_N22_). The variations for GW and DFF were the least was also evident from the distribution. The modal class was coexisting with N22 for PL, DFF, GL, GW, and 100 SW, while for PH, NPT, and FLL the mode was at right of the N22 (**Figure 1** and **Table 1**). The PH and FLL had similar variation (0.16 CV). Interestingly for PL, DFF, and GW, the mean of the mutant garden was equal to the performance of the N22 (23 cm; 93 days; and 2.7 mm). Distributions for all the traits showed that the increase in length mutations were more frequent than increase in size.

**Table 1:** Descriptive statistics of the EMS-induced Nagina 22 mutants for morphological traits, organic constituents, and inorganic content.

	Plant height (cm)	Panicle length (cm)	No. of prod. tillers	Flag leaf length (cm)	Days to 50% flowering (DAS)	Grain length (mm)	Grain width (mm)	100 seed weight (g)	Total Chlorophyll (mg/g)	Wax (mg/g)	Phosphorous (% dry wt.)	Silicon (% dry wt.)	Chlorine (% dry wt.)
Nagina 22	110.00	23.00	8.00	30.00	93	7.6	2.7	1.8	5.45	0.93	0.06	6.87	0.18
C.V.	0.19	0.13	0.61	0.18	0.08	0.11	0.08	0.16	0.11	0.05	0.3	0.24	0.41
Mutants													
Number of mutants	541	541	541	541	541	541	541	541	512	512	84	84	84
Mean	130.77	23.11	9.47	33.11	92.93	8.09	2.68	1.96	5.11	0.87	0.06	6.36	0.22
S.D.	20.78	2.97	4.85	5.33	7.06	0.81	0.20	0.29	0.6	0.05	0.02	1.63	0.07
Range	66.00–190.33	13.67–32.67	3–53	19.00–56.33	61–112	6.02–12.34	1.86–3.41	01–03	2.82–6.83	0.78–0.97	0.03–0.15	1.60–9.13	0.08–0.63
C.V.	0.16	0.13	0.51	0.16	0.08	0.10	0.08	0.15	0.12	0.05	0.32	0.26	0.34
S.E.	0.89	0.13	0.21	0.23	0.3	0.03	0.01	0.01	0.03	0.00	0.00	0.18	0.01


### Performance of the Mutants for DUS Traits and Their Stability

**FIGURE 2:**
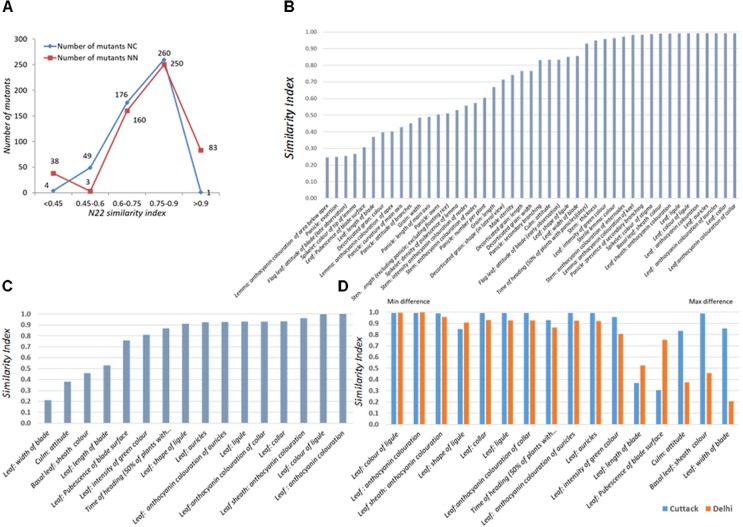
Performance of the rice EMS mutants of Nagina 22 for DUS across two locations, **(A)** Distribution of similarity index of mutants in two locations, New Delhi (NN) and Cuttack (NC); **(B)** Traitwise similarity index at NC; **(C)** Traitwise similarity index at NN; **(D)** Comparison of traitwise similarity index at NC and NN. Similarity scores were assigned by comparing the mutant trait value with the WT trait value. If they are same, a score “1” was assigned; if not “0” score was assigned. The scores were averaged across all the traits to arrive at mutant similarity index. The scores were also averaged over all the mutants for each trait to arrive at trait similarity index.

The set of 541 mutants grown at the NN and NC environments during *Kharif* 2017 was characterized for 15 (all vegetative) and 44 DUS traits, respectively. As 7 and 51 mutants did not germinate, data for 534 and 490 mutants were recorded at NN and NC. Distribution of similarity index across the two environments was similar with only 40–50 mutants showing a WT similarity index less than 0.6 (**Figure 2A**). Under NC and NN, 250 and 260 mutants had similarity index in the range of 0.75–0.9. Under NC, 89.18% of the mutants had similarity index more than 0.60, while at NN, 92.3% of the mutants had similarity index more than 0.6. Both these observations suggested that the mutants were true to type of the WT, N22 except for the mutant phenotype.

The traits that showed maximum variations in the NC environment were anthocyanin coloration of area below apex in lemma, panicle exertion, flag leaf blade attitude (late observation), and color of tip of lemma in the spikelet followed by leaf blade length, decorticated grain color, and pubescence in the leaf surface (**Figure 2B**). These results could not be compared with that of NN environment, since the most varying traits listed earlier are the observations made after the onset of reproductive stage. In the NN environment, leaf blade, culm attitude, and basal leaf sheath color showed more variation, but they were more or less highly stable in the NC environment. Mutants were more variable for leaf blade length and pubescence in the leaf surface in both the environments (**Figures 2B,C**). For the rest of the 11 traits, the index was similar between the two locations, suggesting that the mutants were stable for their phenotype expression (**Figure 2D**).

### Stability of the Mutants Across Environment

**FIGURE 3:**
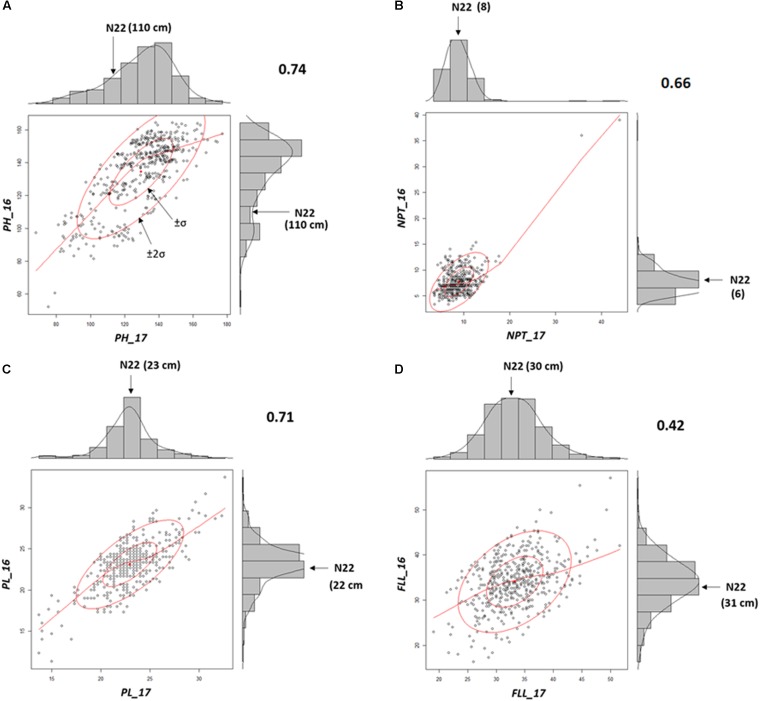
Morphological trait correlations across two years indicating the stability of the mutants **(A)** Plant Height (PH); **(B)** Panicle length (PL); **(C)** Number of productive tillers (NPT); and **(D)** FLL. All correlations were according to Pearson’s linear correlations and were found to be positive and significant at *P* < 0.05.

Mutants grown in NN16 and NN17 were analyzed for the trait correlations across years. Performance of the WT remained nearly equal during both the seasons (PH: 110 cm; PL: 22/23 cm; and FLL: 31/30 cm; **Supplementary Table S3**) with distinct difference in only in NPT, 6 tillers in 2016, and 8 tillers in 2017 (**Figures 3A–D**). As compared with NN17, the mutants had better expression for PL (SD being 2.8 against 2.9 cm) and FLL (SD being 5.8 against 5.1 cm) in NN16 in, but a narrower distribution for PH (SD being 20.9 against 19.6 cm) and NPT (SD being 3.1 against 3.8). Overall, the trait correlations were positive and significant across years, with highest correlation observed for PH (0.74) followed by PL (0.71) and NPT (0.66). The FLL alone showed comparatively low (0.42), but still positive and significant correlation across years.

### Assessment of Genomic Similarity of the Mutants With the WT

**FIGURE 4:**
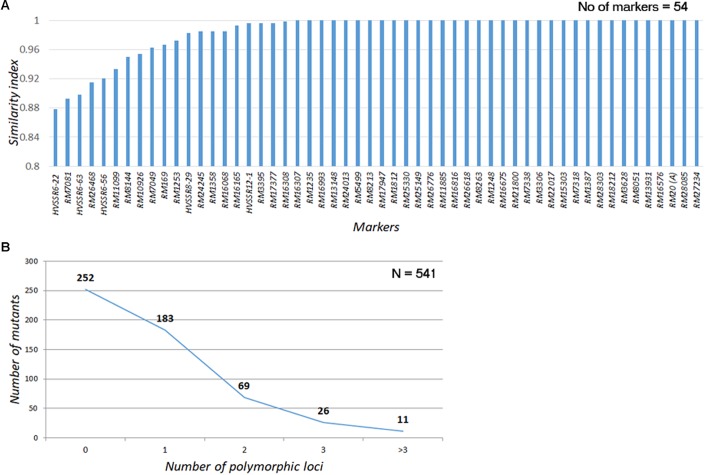
Assessment of genomic similarity of the mutants with Nagina 22 using SSR markers **(A)** Similarity index of SSR markers used for genotyping the mutants; **(B)** Polymorphism in the mutants for SSRs. Similarity scores were assigned by comparing the PCR amplicon size of the mutant with that of WT. If they are same a score “1” was assigned; if not “0” score was assigned. The scores were averaged across all the markers to arrive at mutant similarity index. The scores were also averaged over all the mutants for each marker to arrive at trait marker performance index.

A total of 60 genomewide SSR markers (52 SSRs and 8 HvSSRs; **Supplementary Figure S3A**), which had exhibited appreciable polymorphism with a postgreen revolution-representative mega rice variety, IR 64, were used for assessing the genomic similarity of the mutants with the WT (**Supplementary Figure S3B**). Except for four SSRs, all the other had long direpeat motifs. A total of 54 markers, including 49 SSRs and 5 HvSSRs, provided clear amplification across all the 541 mutants (**Supplementary Figure S3C**). Nearly 81.33% of the mutants (440/541) had amplification across all the loci without fail, while 82 mutants (15.15%) had missing allele call for one of the marker loci. Only 19 mutants (3.5%) had missing data in two or more loci. Thirty-four of the total 54 markers did not show any polymorphism across the mutant garden (**Figure 4A**). Of the 20 markers, only three markers, two HvSSRs, and one SSR (RM7081) showed polymorphism in nearly 10% of the mutants, while 14 showed polymorphism in less than 5% of the mutants. About 46.58% of the mutants (252 out of 541) were completely identical with the WT across all the 54 markers tested, while 183 (33.82%) and 69 (12.75%) mutants showed polymorphism in one and two loci, respectively (**Figure 4B**). Only 37 (6.8%) of the mutants were found to be polymorphic for three or more loci. These results clearly indicated that the mutants were highly similar to N22 in their genetic background. Thus, the entire mutant resource is an excellent material for carrying out forward and reverse genetics.

### Variation in the Mutant Garden for Physiological and Biochemical Traits

**FIGURE 5:**
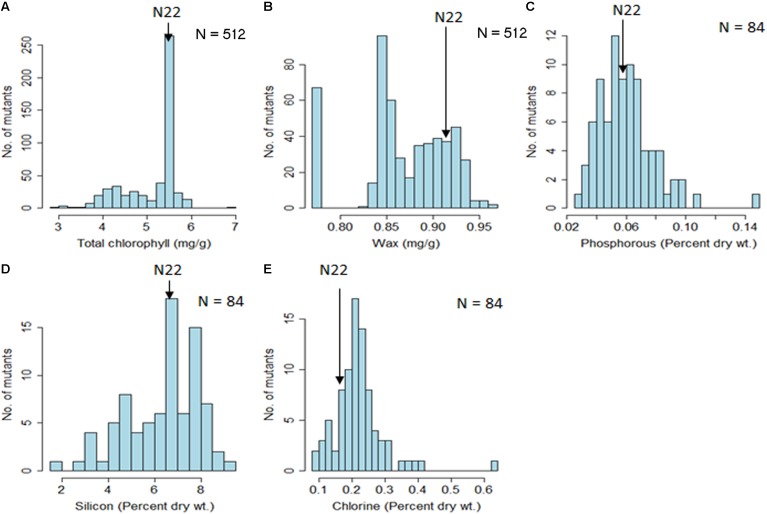
Descriptive statistics and distribution of organic (chlorophyll and wax content) and inorganic constituents (P, Si, and Cl) in the rice EMS mutant garden of Nagina 22. **(A)** Total chlorophyll; **(B)** Epicuticular wax; **(C)** Phosphorous; **(D)** Silicon; **(E)** Chlorine. Units of the traits measured are indicated in the parenthesis. The number of mutants (N) used for each trait assessment is indicated.

For chlorophyll content, it could be clearly seen that the mutants were clustered at and around WT with a clear mode coinciding with the N22 value of 5.45 mg/g of leaf (**Figure 5A** and **Table 1**). This demonstrated the validity of the mutant garden as well as the high-throughput estimation procedures followed. However, wax content did not show such a pattern and showed more of loss of function mutants, with the mutant garden mean (0.87 μg/g) being less than the N22 wax content of 0.93 μg/g (**Figure 5B** and **Table 1**). There was also a distinct difference in the CV in these two traits, with chlorophyll content having a healthy CV of 12%, while wax content had only 5% CV. Among the inorganic constituents, the results had to be interpreted with caution because only 84 mutants randomly selected from the mutant garden were selected for the analysis, and the measurement was only from the one tissue, husk powder, though replicated samples were used for analysis (**Figures 5C–E** and **Table 1**). Further, variation for the three inorganic constituents was very high, in the range of 32% (P), 26% (Si), and 34 % (Cl), and for P, the N22 mean and the mutant mean were the same 0.06% on dry weight basis (**Figure 5C** and **Table 1**). In case of Si, the N22 mean was higher than the mutant mean (6.87 against 6.36% dry weight), while for Cl, it was the other way around with higher Cl content in the mutants (0.22 against 0.18% dry weight).

### Whole Genome Sequencing of the WT and Four Selected Mutants and Development of Reference Genic Sequence

In this workflow, we have created a N22 genic sequence reference assembly based on short read data, as whole genome assembly would require long read data (Nanopore/PacBio) for a reliable assembly. After trimming the adaptor sequences and removing the reads with quality score <30, a total of 120 million high-quality reads of N22 from both HiSeq and MiSeq, having an average read length of 101 and 251 bp, respectively, were used for creating N22 reference genic sequences (**Table 2**). Four different mutants identified for various traits like drought tolerance, grain chalkiness, and SGT were also sequenced, and from this, a total of 458 M high-quality reads were obtained. Since EMS creates point mutations (Till et al., 2007; Parry et al., 2009), we used this as a basis to improve the coverage of the N22 genic sequence data (**Supplementary Figure S5**). We used a CLC Genomics pipeline to identify the consensus base from the mutant reads, fill the gaps in N22, and improve the coverage. The total depth of the rice genome available to create N22 genic reference increased roughly from 48 to 162×, nearly a 357% increase, by using the data from all the four mutants. The increment we obtained with addition of each mutant is shown in **Table 2**. The 66,638 genic sequences, comprising 113.3165 Mbp available in the latest version of the MSU database, were used as reference to create the N22 genic reference. The number of reads that mapped to the genic sequences increased from 50 to 265 M, nearly 500% increase (**Table 2**). Although in our analysis, we have not considered the unmapped reads, which comprised of the reads that mapped to intergenic sequence as well as those that did not map to the Nipponbare reference at all, the proportion of unmapped reads also increased from 70 to 313.5 M. Such an increase, to the tune of 447%, also stand testimony to the huge amount of data generated in the study. Finally, 45.03% of the total reads generated from N22 and the mutants were found to map to the genic reference (**Table 1**). As expected, this statistics did not show a drastic difference upon addition of each mutant data and was in the range of 41–45%, as the proportion of genic sequence in the whole genome is constant. This also showed that the library preparation was optimal across all the samples, and sequencing coverage of the genome was uniform across mutants and the WT.

**Table 2:** Summary of Nagina 22 whole genome sequencing (WGS) and genic sequence mapping.

Details	N22 WGS	N22 + WGS of one mutant	N22 + WGS of two mutants	N22 + WGS of three mutants	N22 + WGS of four mutants
Number of high-quality reads	120781436	162268968	352613804	458853320	578981912
Total number of bases (Gbp)	19.05	22.97	35.03	51.80	64.70
Sequencing depth (X)	47.63	57.43	87.58	129.51	161.75
Number of reads mapped with genic sequences	50657199	69119816	152335647	204870739	265412027
Number of reads mapped with intergenic sequences	70124237	93149152	200278157	253982581	313569885
Number of bases in the genic sequence mapped reads	7843799793	9576152265	17516004445	22725052021	28638120831
Number of bases in the intergenic sequence mapped and unmapped reads	11207197664	13397624028	17516004445	29077911302	36060550009
% of mapped read bases	41.17	41.68	42.48	43.87	45.03


We further estimated how many “N”s (gaps) finally remained in the N22 genic reference assembly created this way (**Table 3**). With N22 data alone, a huge number of gaps accounting for nearly one-third of the genic sequences remained (31.79%). With all the four mutant sequences, only 6% of the data had gaps indicated as “N”s (**Table 3**). Of the 66,638 genes, 2,480 genes were left completely uncovered, while using N22 whole genome resequencing (WGS) data alone, which reduced by nearly 90% and came to just 249 genes after polishing the N22 genic sequence with the mutant data. This was also reflected by the steady increase in the number of genes with different proportions of N less than 50, 75, and 90%. Overall, the number of genes with <50% of “N”s increased from 49,237 to 64,780 (**Table 3**). The improvement was more pronounced while comparing on the basis of <10% “N”s and no “N”s: the number of genes that had more than 90% coverage increased from 17,110 to 55,329 for <10% “N”s, while the complete coverage increased from a mere 4,290 to 27,764 genes, making the resource more meaningful and useful for mapping.

**Table 3:** Nagina 22 reference gene assembly summary before and after gene polishing using mutant data.

Details		Rice MSU.v.7_reference genic sequence	N22 WGS	N22 + WGS of one mutant	N22 + WGS of two mutants	N22 + WGS of three mutants	N22 + WGS of four mutants
Total number of genic sequence		66338	66338	66338	66338	66338	66338
Total number of bases		113316541	77369048	81591225	96951662	103239568	106530223
Number of A		28185154	20498770	21649576	23569852	25968532	26566492
Number of T		27986132	17533364	18465058	20158495	25073446	26163667
Number of G		30149438	19577710	20627359	23654859	27249073	28280777
Number of C		26995746	19759204	20849232	29568456	24948517	25519287
Number of N		71	36021287	31797345	16384936	10102378	6800670
% of N		-	31.79	28.06	14.46	8.92	6.00
Number of	*N* < 50%	66338	49237	52179	58945	63931	64780
genes covered	*N* < 25%	66338	31623	35367	42898	59350	62054
	*N* < 10%	66338	17110	20148	34567	49800	55329
	*N* = 0	-	4290	5641	8695	22687	27764
Number of genes with no reads mapped		-	2480	2053	1605	448	249


### SNP Calling in the Mutants and Their Frequency in the Genic Regions

**FIGURE 6:**
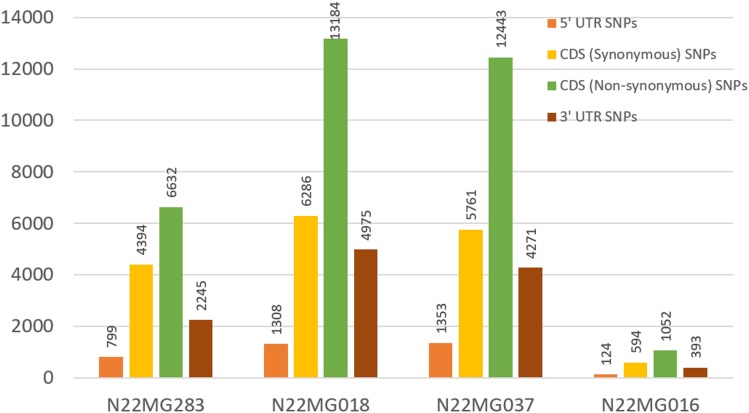
SNP distribution across different genic regions in the mutants.

After establishing the reference genic sequence of N22, the SNPs in the four mutants were directly called instead of following the routine Nipponbare reference-based pipeline. Number of SNPs across the mutants ranged drastically from 2,163 to 25,752 in the genic sequences, with an average of 16,453 SNPs per mutant (**Table 4**). Number of transversions was nearly double than transitions across all the mutants invariably. The distributions of SNPs in the various genic regions revealed more number of SNPs in 3′ UTR region than 5′ UTR (**Figure 6**). Furthermore, 75% of the identified SNPs from the genic region corresponded to the CDS region. The SNPs in the CDS regions were further translated to compare the number of synonymous and non-synonymous amino acid changes created by mutagenesis. We found a huge proportion of non-synonymous changes consistently across all the four mutants ranging from 60.15% (6,632 out of 11,026 SNPs in the CDS region) to 68.35% (12,443 SNPs out of 18,204 in the CDS region) (**Figure 6**). While including the untranslated regions (UTRs) too for calculating the frequency of non-synonymous changes, this was around 47–55.8%. Compared with this, the synonymous changes were only 30%. Frequency of insertions and deletions (InDels) in the four mutants ranged from 898 to 2,595 (**Table 4**), with more than 80% of them being 1–2 bp in size (data not shown). For none of the four mutants, the SSR similarity index was 1, indicating that all of them harbored one or more cryptic variations (**Table 4**). When we looked into the polymorphic SSRs, we found that they harbored 1–3 variations and all of them were from hypervariable regions rather than regular SSR motifs. For instance, N22MG283 was variable for HvSSR6-22, HvSSR6-56, and HvSSR9-7; N22MG018 was variable for HvSSR6-22; N22MG037 was variable for HvSSR6-63; and N22MG016 was variable for HvSSR6-22, HvSSR6-56, and HvSSR6-63.

**Table 4:** SNPs identified in the four mutants and their comparison with the WT for DUS, SSR, and major morphological traits.

Mutant ID	Mutant trait phenotype	DUS similarity index	SSR similarity index	PHT	NPT	PL	FLL	DFF	GL	GW	100 seed weight	Sequencing depth	Transitions	Transversions	Others (*N*->A/C/G/T)	Total number of SNPs	Number of InDels
N22MG283	Drought tolerance	0.68	0.96	134.67	6	25.33	31.67	100	8.90	2.43	2.50	10×	6821	3730	3517	14068	898
N22MG018	Grain size	0.84	0.98	85.67	4	24.00	32.33	98	8.43	2.56	2.51	16.2×	17010	8310	432	25752	2595
N22MG037	Stay Green	0.71	0.98	94.00	9	24.33	26.67	99	7.16	2.64	1.73	10.27×	15769	7662	395	23826	2175
N22MG016	Stay Green	0.60	0.94	84.67	14	23.67	26.33	84	7.70	2.04	1.30	31.66×	1114	718	331	2163	1144
Nagina 22	WT	1.00	1.00	110.00	8	23.00	26.00	93	7.60	2.70	1.80	32.05× (MiSeq)	-	-	-	-	-


*PHT, plant height; NPT, number of productive tillers; PL, panicle length; FLL, flag leaf length; DFF, days to 50% flowering; GL, grain length; GB, grain width.*

### Demonstration of a Reverse Genetic Pipeline for Identification of Causal Mutants

Since the N22 reference gene sequence information was established, we identified SNPs in the candidate genes by a reverse genetic pipeline. We report here the results obtained from two SGT mutants, ascertained by phenotyping under dark-induced senescence. We first shortlisted a set of 81 candidate genes related to SGT such as genes involved in chlorophyll biosynthesis and degradation, cytokinin metabolism-related genes, and other source-sink-related genes and other genes implicated for SGT in literature (**Supplementary Table S4**). We identified a total of 61 SNPs in 24 genes in SGM1 and 69 SNPs distributed across 29 genes in SGM2. Of these, 17 and 12 SNPs created non-synonymous codons in SGM1 and SGM2. Further, one SNP in SGM2 created a stop codon in LOC_Os09g0532000 encoding senescence-inducible chloroplast protein, which activates chlorophyll-degrading pathway during leaf senescence and popularly known as *sgr*. Thus, generating the N22 reference genic sequence and using it for quick reverse genetic approach could identify the causal mutation. No such changes could be identified by the reverse genetic pipeline in SGM1 and, hence, mapping is being planned through forward genetic approaches.

### Mutant Database

**FIGURE 7:**
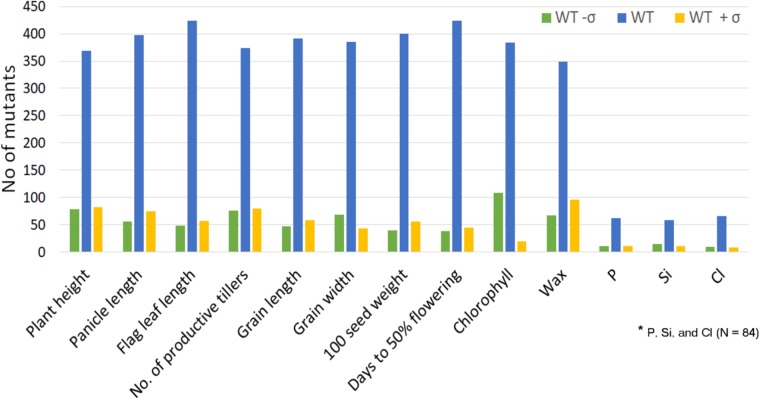
Distribution of the mutants beyond ±1 standard deviation for the morphological traits and organic and inorganic constituents. This is to show that the mutants with extreme trait value are available for each trait.

**FIGURE 8:**
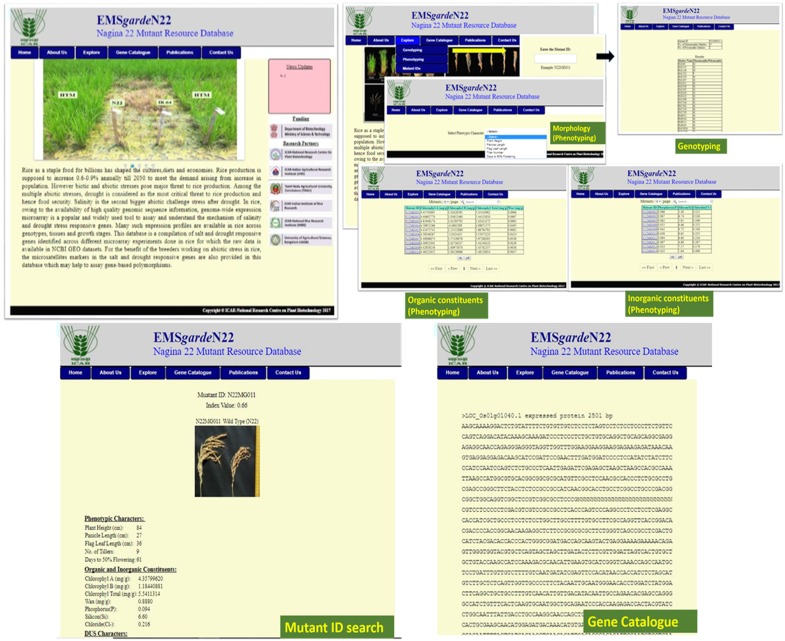
Snapshot of the mutant garden database indicating the available search options.

To provide access to the EMS mutant resources to rice researchers as well as to provide basic information about the resources, a mutant database named “EMS*garde*N22” was developed. The entire dataset presented in this research paper, i.e., data on major morphological variations, chlorophyll and wax content, DUS data, SSR profile data of the entire 541 mutants, and the element content data of the 84 mutants is available in the database. In addition to this, images of the panicle architecture of 493 mutants have also been stored in the database. A search option has been enabled to retrieve information on the extreme mutants of both high and low value, for each trait (**Figures 7, 8**). Once the desired mutants are shortlisted, the genotype and phenotype information of the mutant can be retrieved by submitting the mutant ID as query. A separate tab for retrieving the genic information of N22 in batches has been provided in the database. This information can be downloaded in the FASTA format and used for performing local BLAST with the mutant sequence information. As and when the N22 genic sequence resource is further improved, it will be updated in the DB. Further, there is a plan to provide both the forward (MutMap) and reverse genetic analysis pipelines in the DB.

## Discussion

Induced mutations have the dual distinction of being central to basic sciences, especially in molecular genetic and genomic research, while they can also serve as a donor genetic stock (breeding material) in plant breeding. Both these applications are relevant in a major food as well as genomic model crop, rice, and this is evident from the number of mutant resources developed (Wang et al., 2013; Li et al., 2017), number of important traits and genes characterized from the mutant resources (Plett et al., 2010; Huang et al., 2011; Talla et al., 2016), and mutation breeding-derived products such as herbicide-tolerant rice (US 20070028318 A1; Shoba et al., 2017). Mutant resources developed through different mutagens whether, T-DNA-based or chemical or physical agent, have their own set of advantages and disadvantages (Wang et al., 2013; Amitha Mithra et al., 2016). Considering the survival issues in KO mutants generated by physical mutagens, and the allelic series advantage offered by EMS mutants, we generated a mutant resource in the background of the pregreen revolution variety N22 (Mohapatra et al., 2014) and recently augmented this resource to 87,000 independent M_2_ mutants. In bread wheat, such an EMS mutant resource in the background of a pregreen revolution variety with no known genes for semidwarf stature has been developed (Dhaliwal et al., 2015). Since the genomic constitution of the modern day varieties, bred after 1960s, is completely different from that of the traditional cultivars, mutations in their alleles could be an altogether a new resource from the rest of the resources available. Further, N22 is an important resource for drought tolerance and heat tolerance in rice, and is supported by a large number of QTLs, miRNAs, expression data, and databases, which would be useful in characterizing the mutants (Kumar et al., 2014; Kansal et al., 2015; Mutum et al., 2016; Mangrauthia et al., 2017; Sandhu et al., 2017; Shanmugavadivel et al., 2017).

A set of 541 morphologically distinct mutants reported here could be used for fine genetic studies, while the entire resource can be used for large-scale screening for any trait of interest such as resistance to biotic and abiotic factors, nutrient use, and various organic and inorganic constituents, and further molecular studies. From this resource, already an herbicide-tolerant mutant had been identified and is being used in breeding (Shoba et al., 2017). The entire rice-growing community across the globe relies on the *sd1* gene for the semidwarf nature of rice with a mutation in GA20 oxidase. Identification of 81 dwarf mutants available in the mutant garden (**Figure 8**)^1^ and more than 500 mutants in the entire resource of 87,000 mutants (data not shown) could give us alternate source for reduced PH. One of these 81 mutants had been found to have mutation in *OsCCD7* cosegregating with dwarf stature and very high number of tillers (Kulkarni et al., 2014), while the others are yet to be characterized. Wherever possible, the mutants are being screened in a large scale: for example, the mutants are grown in disease hot spot locations in appropriate design to identify mutants showing resistance to diseases such as rice blast.

Since the EMS mutagen is known to induce multiple point mutations and, at times, cryptic variations, examination of genomic similarity of the mutants with the WT has been recommended and followed by different researchers (Wu et al., 2005; Lima et al., 2015). Hence, we carried out the DNA profiling of all the 541 mutants across 54 genomewide markers. It is well established in rice that the average SSR marker polymorphism between any two varieties is around 12–15%, with most of the SSRs being of conserved nature, although polymorphism as low as 11.2% to as high as 63.9% have been reported (Govindaraj et al., 2005; Yoon et al., 2006; Shanmugavadivel et al., 2013). Hence, while selecting the markers for genomic similarity analysis, we ascertained that the marker loci are not just chosen randomly based on their chromosomal distribution, but also their polymorphism with an established variety from a different background such as IR64. Further, we also studied 44 DUS characters to assess the similarity of the mutants which, to our knowledge, is being done for the first time for mutants, though this is a regular exercise in varietal testing (Shobha Rani et al., 2004; Mondal et al., 2014). From the DUS and genomewide marker data, the resource has been established as the true mutant resource of N22. Further, these mutants also have been established as stable ones, from their high similarity indices across locations and that they had been advanced to M_3_–M_8_ generations.

From the first sequencing-based approach for mapping the EMS-induced mutants, it was known that EMS induces 900–2,000 mutations in rice (Abe et al., 2012). However, our efforts in WGS of the mutants revealed that the frequency of the mutation was nearly 10-fold higher with a frequency of nearly 2,163–25,753 SNPs and 898–2595 InDels in the genic sequences and, especially, CDS region, with an average of 16,453 SNPs and 1,703 InDels per mutant. Extrapolating this to the entire 541 mutants gives rise to nearly one mutant for every 77 kbp with nearly 50% of them leading to non-synonymous codons (**Figure 6**). Such a higher range of mutations by EMS, in the range 1/12 to 1/47 kbp, have been reported in wheat, but not in rice (Slade et al., 2005; Chen et al., 2012). Despite the presence of these many SNPs, we did not see drastic changes in phenotypes, with their phenotype changes restricted to one or two morphological variations (**Table 4**). This was supported by high similarity indices across DUS and SSR, suggesting the presence of very high phenotype plasticity in the mutants. We expect such a large number of SNPs to have serious implications in mapping the mutants, since meiosis will not be able to recombine such a high number of mutations by F_2_ generation, which would essentially need a large number of homozygous mutant type individuals (from the segregating generation) to be pooled for sequencing and this, in turn, would escalate the cost of sequencing and the entire mapping experiment.

In the T-DNA-, transposon-, and retroposons-based insertional mutants, it is common to develop a database with the information on flanking sites, segregation data, and markers (Jeon et al., 2000; Miyao et al., 2003; Li et al., 2007). In the fast neutron-based mutants also, a genome browser named, kitbase, has recently been developed (Li et al., 2017). Such a characterization is not possible with EMS-induced mutants: (i) owing to lack of deletions and insertions and (ii) that developing a genome browser would need sequencing in comparatively more depth owing to their high mutation frequencies (Slade et al., 2005; Chen et al., 2012). Still, we ultimately aim to characterize the mutants thoroughly for all possible phenotypes and make them available to the entire research community. In this endeavor, a mutant database has been developed, where we could update the characterization with as many parameters as possible and have made a modest beginning with major morphological variations, 44 DUS traits, a few organic and inorganic constituents, and images for panicle architecture.

Since the ultimate aim in developing any mutant resource is to assign functions to genes, we have initiated efforts toward integrating forward and reverse genetic approaches. In doing so, we have made effective use of the N22 and mutant WGS data generated by us and ultimately hosted 27,764 genes of the total 66,338 rice genes with the complete sequence information and further a set of 27565 genes with just <10% gaps, making the resource useful for mapping. We have also demonstrated the effective use of the resource by identifying the causal mutation in one of the stay-green mutants (SGM1) as the already known loss of function allele of the SGR2 gene.

## Conclusion and Future Prospects

The mutant resource generated in the Nagina 22 background in rice along with the related resources such as mutant database, improved phenotype characterization, and the reference sequence of N22 is expected to serve the rice research community in identification of novel genes and alleles and their characterization. Screening of the resources for important traits such as nutrient use efficiency, resistance to disease such as sheath blight/blast/bacterial blight, and photosynthetic efficiency by using high-throughput techniques, such as imaging, will give us genes that can be deployed in rice improvement programs.

## Author Contributions

AMVS and RPS conceived this work. PK and PBK carried out DUS and morphological characterization at Delhi location, while MB and PS did it in Cuttack location, MR and NY carried out biochemical and wax characterizations. RD carried out inorganic constituent characterization. Selection of SSRs was by AMVS. SSR genotyping and allele calling was by PK, PBK, MR, and NY. Genic sequence pipeline and analysis was by AM and AMVS. SV and AMVS designed and developed the database. JP, MK, MS, RM, SN, SS, GS, AKS, NS, and TM generated the resource, stabilized it over generations, and maintained it. CP and AMVS carried out the data analysis and prepared the figures and tables. AMVS and GS wrote the manuscript. AMVS coordinated and supervised the entire work. Everyone has read the manuscript, made substantial, direct, and intellectual contribution to the work, and approved it for publication.

## Conflict of Interest Statement

The authors declare that the research was conducted in the absence of any commercial or financial relationships that could be construed as a potential conflict of interest. The reviewer YO and handling Editor declared their shared affiliation.

## Footnotes

1 http://14.139.229.201/EMSgardeN22

## Supplementary Material

FIGURE S1 Schematic representation of generation of mutant resources and the phenotyping of 541 mutants.

FIGURE S2 Generation **(A)**, maintenance **(B–D)**, and storage **(E,F)** of the EMS mutant resources of Nagina 22.

FIGURE S3 **(A)** Chromosome wise distribution of SSR markers chosen for assessment of genomic similarity of the mutants with Nagina 22; **(B)** Polymorphism survey of the WT, Nagina 22 with a indica variety IR 64; **(C)**: Genotyping of the mutants with HvSSR 8-29 and with HvSSR 6-56.

FIGURE S4 Schematic illustration of the mutant database architecture.

FIGURE S5 Schematic representation of the development of Nagina 22 genic sequence resource using the whole genome sequencing data of Nagina 22 and four mutants. **(A)** Flow chart of the sequence development; **(B)** methodology adopted to assemble polished Nagina 22 sequence.

TABLE S1 Mutant resources available in rice across the globe.

TABLE S2 Details of the SSR primers used for assessment of genomic similarity of the mutants with Nagina 22. HvSSRs are indicated in green font.

TABLE S3 Descriptive statistics of major morphological traits in the rice EMS mutant garden of Nagina 22 in *Kharif* 2016 and *Kharif* 2017 at New Delhi.

TABLE S4 List of 81 genes used for reverse genetic mapping of the stay-green trait mutants.
