# Supplementary figures and images for: Whole Genome Characterization of a Few EMS-Induced Mutants of Upland Rice Variety Nagina 22 Reveals a Staggeringly High Frequency of SNPs Which Show High Phenotypic Plasticity Towards the Wild-Type
Source: Front Plant Sci. 2018 Sep 4;9:1179. doi: 10.3389/fpls.2018.01179 (PMC6132179; doi:10.3389/fpls.2018.01179)

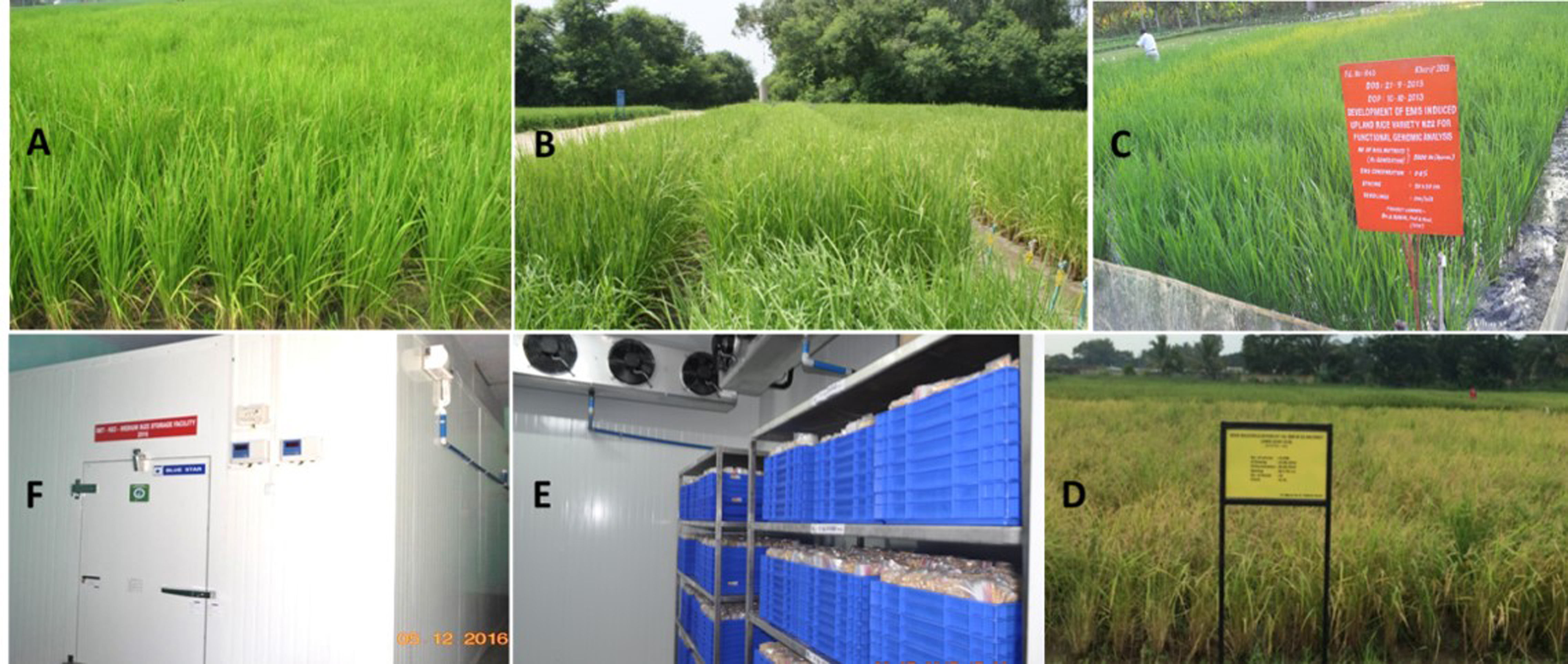

Supplement: FIGURE S2 — Generation (A), maintenance (B–D), and storage (E,F) of the EMS mutant resources of Nagina 22. [file Image_2.JPEG]

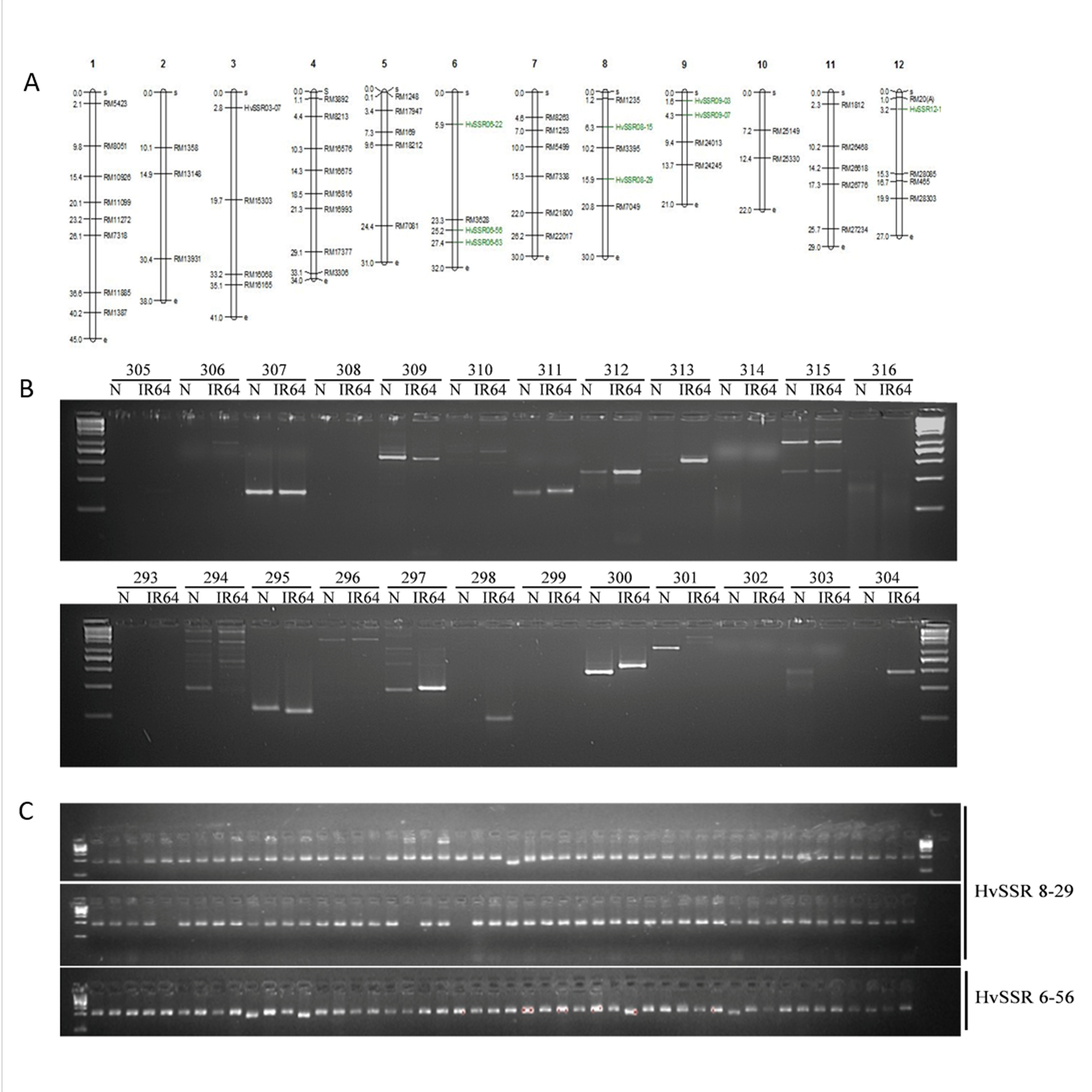

Supplement: FIGURE S3 — (A) Chromosome wise distribution of SSR markers chosen for assessment of genomic similarity of the mutants with Nagina 22; (B) Polymorphism survey of the WT, Nagina 22 with a indica variety IR 64; (C): Genotyping of the mutants with HvSSR 8-29 and with HvSSR 6-56. [file Image_3.TIF]

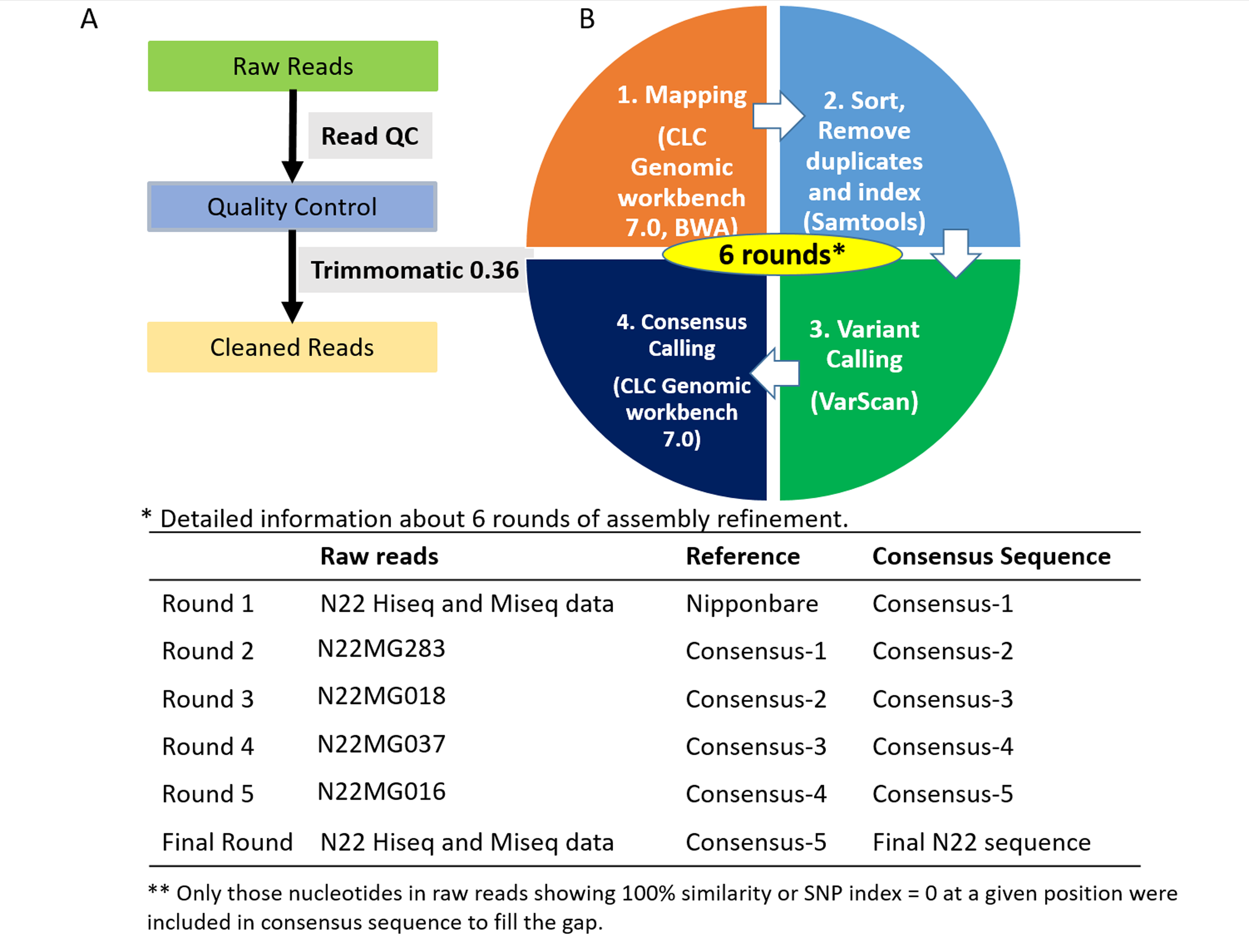

Supplement: FIGURE S5 — Schematic representation of the development of Nagina 22 genic sequence resource using the whole genome sequencing data of Nagina 22 and four mutants. (A) Flow chart of the sequence development; (B) methodology adopted to assemble polished Nagina 22 sequence. [file Image_5.TIF]
