# Supplementary material for: Whole Genome Characterization of a Few EMS-Induced Mutants of Upland Rice Variety Nagina 22 Reveals a Staggeringly High Frequency of SNPs Which Show High Phenotypic Plasticity Towards the Wild-Type
Source: Front Plant Sci. 2018 Sep 4;9:1179. doi: 10.3389/fpls.2018.01179 (PMC6132179; doi:10.3389/fpls.2018.01179)
Supplement: TABLE S1 — Mutant resources available in rice across the globe. [file Table_1.DOCX]

**Supplementary Table 1:** Mutant resources available in rice across the globe

| **SN** | **Genetic Background** | **Mutagen** | **No o lines** | **Mutant resources** | **Institute** | **Reference** |
| --- | --- | --- | --- | --- | --- | --- |
| 1 | Dongjin, Hwayoung Kitaake | T-DNA (GT, AT) | 58943 | POSTECH Rice Insertion Database (RISD) | Pohang University of Technology and Kyung Hee University, Korea | Jeon, et al., 2000. |
| 2 | Zhonghua 11 Zhonghua 15 Nipponbare | T-DNA (ET) Tos17 | ~129000 | Rice Mutant Database (RMD) | Huazhong Agricultural Univ, Zhejiang University, China | Wu, et al., 2003  Zhang, et al., 2006 |
| 3 | Tainung 67 | T-DNA (AT) | 45126 | Taiwan Rice Insertion Mutant (TRIM) | Academia Sinica, Taiwan | Hsing, et al., 2007  Wu, et al., 2017 |
| 4 | Nipponbare | T-DNA (ET) (+Ds) Tos17 | 27832 | Oryza Tag Line (OTL) Génoplante | CIRAD-INRA-IRD-CNRS, France | Lorieux, et al., 2012  Wu, et al., 2017 |
| 5 | Zhonghua 11 | T-DNA (ET) | 12614 | Shanghai Insertion Population (ShIP) | SIPPE, China | Fu, et al., 2009 |
| 6 | Nipponbare | T-DNA (AT) | - | Chinese Academy of Agricultural Sciences (CAAS) | Chinese Academy of Agricultural Sciences (CAAS), Beijing | Wei, et al. 2013 |
| 7 | Nipponbare | Tos17 | 47196 | Tos17 insertion database (NIAS) | National Institute of Agrobiological Sciences, Japan | Wei, et al. 2013 |
| 8 | Nipponbare | Ac/Ds, (GT/ET) | - | CSIRO | CCSIRO plant Industry, Australia | Eamens, et al., 2004 |
| 9 | Nipponbare | Ac/Ds (ET) | 1300 | EU-OSTID | European Consortium | Van Enckvort, et al., 2005 |
| 10 | Nipponbare | Ac/Ds (GT) | 2000 | Temasek Ds | Temasek Life Sciences, Singapore | Wei, et al. 2013 |
| 11 | Nipponbare | Ac/Ds (GT) En/Spm Ac/Ds AT |  | UC Davis (UCD) Ac/Ds and En/Spm populations | University of California, Davis, USA | Kolesnik, et al., 2004 |
| 12 | Dongjin  Byeo | Ac/Ds (GT) | 4820 | Plant Molecular Biology & Biotechnology Research Center (PMBBRC), Korea | Plant Molecular Biology & Biotechnology Research Center, Korea | Kim, et al., 2004 |
| 13 | Nipponbare | Ac/Ds |  | Cornell University | Cornell University, USA | He, et al., 2007 |
| 14 | Nipponbare | Ds insertion  Ds transposants | 175 | - | National University of Singapore, Singapore, Singapore | Ramamoorthy, et al., 2011 |
| 15 | Nipponbare | RNAi mutant library | 211 | - | Resources and Genetic Improvement Chinese Academy of Agricultural Sciences Beijing China | Wang, et al., 2013 |
| 16 | IR64 | Zinc finger nucleases | - | - | International Rice Research Institute, Metro Manila, Philippines | Cantos, et al., 2014 |
| 17 | Arabidopsis  (Overexpressing Rice cDNA) | - | 17,985 | RiceFOX | Plant Science Center, 1-7-22 Suehiro-cho, Tsurumi-ku, Yokohama, 230-0045 Japan | Sakurai, et al., 2010 |
| 18 | Kitaake | Fast neutron | 1504 | - | University of California, Davis, CA 95616, USA | Li, et al., 2017 |
| 19 | Nipponbare | γ-ray ion beam | 34844 | National Institute for Agrobiological Sciences (NIAS) mutant population | National Institute for Agrobiological Sciences, Japan | Miyao, et al., 2003 |
| 20 | IR64 | Fast neutron γ-ray, DEB, EMS | 60,000 | IR64 deletion mutant population | International Rice Research Institute, The Philippines | Wu, et al., 2005 |
| 21 | Kasalath SSBM | γ-ray, EMS | 1000 | Zhejiang mutant population | Zhejiang University, China | Chen, et al., 2003 |
| 22 | TC65, Yukihikari Kitaano, Kinmaze | MNU, EMS | 9565 | Oryzabase | National Institute of Genetics, Japan | Kurata, et al., 2006 |
| 23 | Tainung67, IR64 | SA EMS | - | Taiwan Agriculture Research Institute (TARI) | Taiwan Agriculture Research Institute, Taiwan | Wei, et al., 2013 |
| 24 | Nipponbare | SA, MNU | - | UC Davis (UCD) TILLING population | University of California, Davis, USA | Till, et al., 2007 |
| 25 | BRS Querencia | EMS | 340 (M3) | Plant Genomics and Breeding Center | Plant Genomics and Breeding Center, Brazil | Luz, et al, 2016 |

**Supplementary Table 2**: Details of the SSR and HvSSR primers used for assessment of genomic similarity of the mutants with Nagina 22

| **Primer ID** | **Motif** | **Repeats** | **Chromosome** | **Forward Primer** | **Bases** | **Reverse Primer** | **Bases** | **Expected amplicon** | **SSR start** | **SSR end** |
| --- | --- | --- | --- | --- | --- | --- | --- | --- | --- | --- |
| RM5423 | AG | 16 | 1 | TCCCACTTGCAGACGTAGGTAGG | 23 | CACTGATCTGATGCAACTGTTTGG | 24 | 147 | 2169222 | 2169253 |
| RM8051,  RM8045,  RM572 | AG | 14 | 1 | CGCGGTTAATGTCATCTGATTGG | 23 | CCATACTTCGAGATCCAAGACTGACC | 26 | 168 | 9865594 | 9865621 |
| RM10926 | AG | 10 | 1 | CCCACTCTTGCAGCAGTTCTCG | 22 | GTCAGACTCGTCGCCACTTCACC | 23 | 148 | 15378856 | 15378875 |
| RM11099 | AT | 43 | 1 | CTAGGTCTGTACACGCACTGAAGG | 24 | CAAATAGCTCGACACAGGAGATCG | 24 | 266 | 20059015 | 20059100 |
| RM8144 | AT | 31 | 1 | TAGGGTGTCCTATCAGGTGAGG | 22 | TTACTACATAGCTACTCCCTCTGTCC | 26 | 265 | 23206225 | 23206286 |
| RM7318 | ACAT | 6 | 1 | GATTCCATAGCTGCGTGATTCTCC | 24 | AGATGAAACTGGCACGTGTGTCG | 23 | 107 | 26136814 | 26136837 |
| RM11885 | AG | 13 | 1 | ACACCCTTCATTTGCGTGTATGG | 23 | GGCACCTTCGACTTTGTAAATGC | 23 | 180 | 36680970 | 36680995 |
| RM1387 | AG | 44 | 1 | GCTACCATGCACGAAACAGAGACG | 24 | ACCAAATGTGGCTGGCTGATCG | 22 | 195 | 40201060 | 40201147 |
| RM1358 | AG | 24 | 2 | AAAGGTGTGTGATGGCTTGTGG | 22 | AGGGCTCTACTATGCACCAATGC | 23 | 374 | 10184719 | 10184766 |
| RM13148 | AT | 14 | 2 | CATGCAGGTGTTCAAAGATTCG | 22 | TAAACCCAACATGTTCCTCTGC | 22 | 517 | 14927502 | 14927529 |
| RM13931 | AT | 28 | 2 | CGTGTGTTGCAATGAGTGAAAGC | 23 | GAGACGATCGAACTCCTGACTGC | 23 | 226 | 30483707 | 30483762 |
| RM15303 | AAG | 15 | 3 | GAATCGGGTCTACGGTTTAGG | 21 | AAAGGAAGAGAAGAGGCAACG | 21 | 200 | 19700251 | 19700295 |
| RM16068 | AT | 14 | 3 | GATGTCAGTTGTGTGAGTCATTCG | 24 | GAATGGATGGACGGTAGATGG | 21 | 376 | 33192874 | 33192901 |
| RM16165 | AC | 12 | 3 | GGTTAACCAAGAGGAAAGGAACC | 23 | GCTTTGCAACTCACTGTTGTTACG | 24 | 265 | 35047947 | 35047970 |
| RM3892 | AT | 33 | 4 | TCTCTCCCTCTCTGAAATGATCC | 23 | CCACATCTCAAGTAGCTAAACTGC | 24 | 166 | 1123512 | 1123577 |
| RM3892 | AC | 14 | 4 | TCTCTCCCTCTCTGAAATGATCC |  | CCACATCTCAAGTAGCTAAACTGC |  | 166 | 1123577 | 1123604 |
| RM8213 | AG | 19 | 4 | TGTTGGGTGGGTAAAGTAGATGC | 23 | CCCAGTGATACAAAGATGAGTTGG | 24 | 179 | 4418222 | 4418259 |
| RM16576 | AT | 13 | 4 | AAGACTTGGTGTATGGTGTGTGG | 23 | CGGCAGTGGGTGTCTAAGTACC | 22 | 244 | 10368520 | 10368545 |
| RM16675 | ACG | 7 | 4 | AAGACCAACGCCATCTTCTTGG | 22 | GTTCCCGGAGTTGGACAAACC | 21 | 278 | 14317067 | 14317087 |
| RM16816 | AG | 22 | 4 | AACCACCACAGCCTCGCTTTGC | 22 | TTCCACCTCTTCTCACGTCGTTGG | 24 | 260 | 18543061 | 18543104 |
| RM16993 | AC | 16 | 4 | GTCACTCACTCACACACTCTCATCC | 25 | TGGTGGTTCTCTGCTTAATCTGG | 23 | 374 | 21331230 | 21331261 |
| RM17377 | AG | 25 | 4 | ATATTACTTCGACGCTGGATCAGG | 24 | GTCAGTTCGTCAGGCACAACG | 21 | 169 | 29123497 | 29123546 |
| RM3306 | AG | 14 | 4 | AGAGCTCAACGGAGGAAGAAGG | 22 | AAATAGACCTGCTTGCCACAATCC | 24 | 286 | 33106463 | 33106490 |
| RM1248 | AG | 15 | 5 | CTCTCAGGTGTGTTGTACATTGTTCC | 26 | CTGCTCAAACAAGCAGCTAATGG | 23 | 260 | 62666 | 62695 |
| RM17947 | AG | 17 | 5 | GTTCTTGCCTTCTCGGCATGTGG | 23 | GGTCACTGATTCCACCATTCACACC | 25 | 188 | 3442245 | 3442278 |
| RM169 | AG | 12 | 5 | CACCTCCTCCAAGATCCTTATGC | 23 | CTCTCTGTCTCGCTGTCTGTTGC | 23 | 144 | 7397690 | 7397713 |
| RM18212 | AT | 25 | 5 | TAGATGTCAGTGGTCAGTACAGG | 23 | TGAACTAGTACTCCATCCAACC | 22 | 368 | 9610587 | 9610636 |
| RM7081 | AACC | 9 | 5 | CTTCCCGCACTACACTGCACTCC | 23 | CTGCAACTTGCTCATGGAGTTGG | 23 | 97 | 24441878 | 24441913 |
| RM3628 | AG | 13 | 6 | GCCCTAGACACACCCGTACC | 20 | TGCCAGATCAGAAATCATGC | 20 | 91 | 23354455 | 23354480 |
| RM8263 | AG | 13 | 7 | TACGTTACGGTTCACTTCATGG | 22 | GGCCAAGACAACTTCAAGACC | 21 | 390 | 4688095 | 4688120 |
| RM1253 | AG | 16 | 7 | GTTCCCGGAGATGGACTACTCG | 22 | GCACAACATGGTGAACTTGATCC | 23 | 533 | 7001046 | 7001077 |
| RM5499 | AG | 25 | 7 | GGACGAAAGGGTATTTGATTGG | 22 | CCTCAAGGTGGTCTCCTTCTCC | 22 | 194 | 10020417 | 10020466 |
| RM7338 | ATCC | 9 | 7 | CGCATGGATCAATCAATAGTGG | 22 | CAAGTGCTGCTACTCTGTCTCTTGG | 25 | 195 | 15335246 | 15335281 |
| RM21800 | AT | 15 | 7 | GTGAAATTTGCCTCGCTGTAACG | 23 | CATCTAACCCTGCTTTGGACTGG | 23 | 360 | 22001087 | 22001116 |
| RM22017 | AT | 23 | 7 | GCTACCATGCTAGTCGTAATGC | 22 | GCTAGTGTAAACTTTGGCATCG | 22 | 361 | 26221546 | 26221591 |
| RM1235 | AG | 10 | 8 | GAGAAACACAATCAGTGACACC | 22 | CTGAAATTGCACTTCACTGG | 20 | 448 | 1203431 | 1203450 |
| RM3395 | AG | 17 | 8 | CTTGGGAAACTTCACCTCATGG | 22 | CTGAGAGAAGCCACAGGATTAATGG | 25 | 286 | 10288496 | 10288529 |
| RM7049 | AAAT | 11 | 8 | GTGGATCAAACGCAGCTAATAACC | 24 | TGAGTTGAGCAAACGTCTGTTGG | 23 | 137 | 20807910 | 20807953 |
| RM24013 | AG | 12 | 9 | TCCATCTTCCTCTCCTAGAGCTTCC | 25 | CTCCCTGTCCCGAGTTAGTGC | 21 | 193 | 9397477 | 9397500 |
| RM24245 | AG | 16 | 9 | GCTCTGCCCATTGTCCTCTAGC | 22 | AATAGGGCATGTCAGGTCAAAGC | 23 | 176 | 13707394 | 13707425 |
| RM25149 | AT | 33 | 10 | CATGCCTATTGCCTCTGATGACC | 23 | TTGCCCGTAGAACTATTGGATCG | 23 | 317 | 7188929 | 7188994 |
| RM25330 | AG | 17 | 10 | ATCCAGCCACTGGTAGTCACAATACG | 26 | ATGAGGAGGCGCAGGATCACC | 21 | 405 | 12451257 | 12451290 |
| RM1812 | AT | 16 | 11 | CCTACCTCCAGTGAGAGCTAACC | 23 | ACGTGCATTTGTGTGGTTTAGG | 22 | 265 | 2392086 | 2392117 |
| RM26468 | AT | 15 | 11 | ATGTTGGAACAGCCGGAAGG | 20 | GACGAACGCGATAGTTCGAAGG | 22 | 288 | 10272143 | 10272172 |
| RM26618 | ATC | 12 | 11 | AATGGCCCAGGTTATGTTGC | 20 | AGCTAACTGGCCTGAACATACTACC | 25 | 389 | 14239276 | 14239311 |
| RM26776 | AT | 33 | 11 | GTACCAGCGTGGACACAATATTCC | 24 | GAGATGTCATGGTATGGCTTTCC | 23 | 444 | 17309082 | 17309147 |
| RM27234 | AG | 12 | 11 | GCTCCACTTTCCTTCATTCTGAATCC | 26 | GATGAAACCTGACCAGGACAAGC | 23 | 189 | 25702706 | 25702729 |
| RM20(A) | AAT | 26 | 12 | TGTATGCACAGCTGCTCTACTCC | 23 | GCACGACCAGAAATTAACAAGG | 22 | 421 | 970631 | 970708 |
| RM28085 | AT | 23 | 12 | CCCGCTGCAGCAGTTTATTGAGG | 23 | GATCTGGTACCTGCATGGGTTGC | 23 | 388 | 15384479 | 15384524 |
| RM465 | CAT | 12 | 12 | GTGCCTCCATCATCATCATC | 20 | TAGGACAAGCGAAGAAACCG | 20 | 212 | 16750640 | 16750851 |
| RM28303 | AT | 33 | 12 | AGGACTTAAGGCGTCGAAAGATAGC | 25 | CTAGCTGGGTTGGTGTTCTCTAGG | 24 | 465 | 19976304 | 19976369 |
| HvSSR12-11 | AAT | 70 | 12 | TTGGTATTGTTATGTGCAGG | 20 | AAAGCCAACCATGTTTATTG | 20 | 376 | 3284181 | 3284557 |
| HvSSR08-29 | GA | 66 | 8 | AACTGAGAGGCTGCTTGTAT | 20 | TAAAGGGTTCACTCATGGAC | 20 | 325 | 15308050 | 15308375 |
| HvSSR06-56 | CT | 66 | 6 | AGCATTTGTGTGTGCAATAG | 20 | ATGCTTGCCTCATCAGTAGT | 20 | 351 | 24321014 | 24321365 |
| HvSSR06-22 | TGT | 51 | 6 | TTCATTTGGTGTCAGAATCA | 20 | AATTAATCACATCTGCCCAC | 20 | 334 | 5237589 | 5237923 |
| HvSSR06-63 | AT | 68 | 6 | TATGGAGTGTTGTGCATGTT | 20 | GAGGATGTCTTGAGCTTGTC | 20 | 318 | 27111555 | 27111873 |

**Supplementary Table 3:** Descriptive statistics of mutants sown in Kharif season 2016 and 2017 at ICAR-IARI farm

|  | Plant height | | Panicle length | | Tiller numbers | | Flag leaf length | |
| --- | --- | --- | --- | --- | --- | --- | --- | --- |
|  | 2016 | 2017 | 2016 | 2017 | 2016 | 2017 | 2016 | 2017 |
| **N22** | 110 | 110 | 22 | 23 | 6 | 8 | 31 | 30 |
| **CV** | 0.19 | 0.18 | 0.13 | 0.12 | 0.52 | 0.47 | 0.19 | 0.17 |
| ***Mutants*** |  |  |  |  |  |  |  |  |
| **Mean** | 134.0 | 129.2 | 23.0 | 22.9 | 7.8 | 9.2 | 34.1 | 33.0 |
| **SD** | 20.9 | 19.6 | 2.8 | 2.9 | 3.1 | 3.8 | 5.8 | 5.1 |
| **Range** | 52 - 164.33 | 66 - 177.33 | 11.33 - 33.66 | 13.66 - 32.66 | 3.33 - 44.66 | 3.33 - 53 | 16.33 - 57 | 19 - 51.66 |
| **CV** | 0.16 | 0.15 | 0.12 | 0.12 | 0.40 | 0.41 | 0.17 | 0.15 |
| **SE** | 1.0 | 0.9 | 0.1 | 0.1 | 0.1 | 0.2 | 0.3 | 0.2 |

**Supplementary Table 4**: List of 81 genes used for reverse genetic mapping of the stay green trait mutants

| **SN** | **RAP_ID** | **Annotation** |
| --- | --- | --- |
| 1 | Os01g0160100 | Similar to Pyruvate decarboxylase isozyme 2 |
| 2 | Os01g0197700 | Similar to Cytokinin dehydrogenase 2 |
| 3 | Os01g0227100 | Chlorophyll b reductase, Leaf senescence |
| 4 | Os01g0237500 | NmrA-like family protein |
| 5 | Os01g0237750 | Hypothetical conserved gene |
| 6 | Os01g0369700 | Similar to Glutathione S-transferase GST 8 |
| 7 | Os01g0371500 | Similar to Glutathione-S-transferase 19E50 |
| 8 | Os01g0501800 | Similar to Photosystem II oxygen-evolving complex protein 1 |
| 9 | Os01g0652300 | Similar to Phospholipase A1-II 7 |
| 10 | Os01g0742500 | Similar to Hexokinase |
| 11 | Os01g0859300 | Similar to ABA response element binding factor |
| 12 | Os01g0930400 | Potassium transporter, High-affinity K acquisition, Root-to-shoot K transport, K-regulated salt tolerance |
| 13 | Os01g0949800 | Similar to Glutathione S-transferase GST 28 |
| 14 | Os01g0950000 | Similar to Glutathione S-transferase GST 28 |
| 15 | Os01g0952500 | A-type response regulator, Cytokinin signaling |
| 16 | Os02g0117800 | Similar to APG5 |
| 17 | Os02g0274900 | Major facilitator superfamily protein |
| 18 | Os02g0550600 | Plant neutral invertase family protein |
| 19 | Os02g0553200 | Thylakoid membrane-bound ascorbate peroxidase, Tolerance to bacterial blight, Response to NaCl |
| 20 | Os02g0635200 | Similar to Nitrilase 2 |
| 21 | Os02g0771600 | Similar to 1-aminocyclopropane-1-carboxylate oxidase |
| 22 | Os02g0819400 | Phytic acid metabolism |
| 23 | Os03g0126000 | Similar to Phosphorybosyl anthranilate transferase 1 |
| 24 | Os03g0146400 | Pheophorbide a oxygenase, Leaf senescence, Wound responses |
| 25 | Os03g0184550 | Similar to Dihydroflavonol-4-reductase |
| 26 | Os03g0212800 | Similar to Beta-glucosidase |
| 27 | Os03g0324200 | Transcription factor involved in the ethylene signal transduction pathway, Positive regulator of ethylene response, Wound signaling |
| 28 | Os03g0327800 | NAC Family transcriptional activator, Abiotic stress response, Positive regulator of leaf senescence |
| 29 | Os03g0643300 | Ornithine delta-aminotransferase, Abiotic stress tolerance |
| 30 | Os03g0654600 | Similar to Myb-like DNA-binding domain, SHAQKYF class family protein, expressed. |
| 31 | Os04g0206300 | Similar to OSIGBa0147O06.5 protein |
| 32 | Os04g0244400 | Thioredoxin fold domain containing protein |
| 33 | Os04g0442300 | A-type response regulator, Cytokinin signaling |
| 34 | Os04g0457000 | Similar to Chlorophyll a/b-binding protein CP24, photosystem II |
| 35 | Os04g0490000 | Similar to Glutamate synthase [NADH], chloroplast precursor |
| 36 | Os04g0490100 | Similar to protoporphyrinogen IX oxidase |
| 37 | Os04g0543900 | Similar to glutamic dehydrogenase1 |
| 38 | Os04g0581100 | 2OG-Fe |
| 39 | Os04g0682000 | Similar to Autophagy 4a |
| 40 | Os04g0692600 | Staygreen protein domain containing protein |
| 41 | Os05g0137200 | Similar to P-glycoprotein ABCB5 |
| 42 | Os05g0209600 | Similar to Esterase |
| 43 | Os05g0449500 | Component of the SCF E3 ubiquitin ligase complex, Jasmonate-regulated defense responses, Regulation of leaf senescence |
| 44 | Os06g0130400 | ACC synthase, Protein homologous to aminotransferase, Ethylene biosynthesis, Control of starch grain size in rice endosperm |
| 45 | Os06g0157000 | Esterase, SGNH hydrolase-type domain containing protein |
| 46 | Os06g0354700 | Alpha/beta hydrolase-fold family protein, Chlorophyll degradation during senescence |
| 47 | Os06g0632200 | Similar to tryptophan synthase-related |
| 48 | Os06g0667600 | Similar to Glycine decarboxylase complex H-protein |
| 49 | Os07g0141400 | Similar to 23 kDa polypeptide of photosystem II |
| 50 | Os07g0182100 | Similar to Tryptophan synthase alpha chain |
| 51 | Os07g0512200 | Similar to Symbiosis-related like protein |
| 52 | Os07g0556200 | Rieske [2Fe-2S] region domain containing protein |
| 53 | Os07g0558400 | Similar to Chlorophyll a/b-binding protein CP29 precursor |
| 54 | Os07g0658400 | Ferredoxin-dependent glutamate synthase, Leaf senescence and nitrogen remobilization |
| 55 | Os08g0465800 | Similar to Glutamate decarboxylase |
| 56 | Os08g0472800 | Cytochrome P450 family protein |
| 57 | Os09g0255400 | Similar to Indole-3-glycerol phosphate synthase, chloroplast precursor |
| 58 | Os09g0346500 | Similar to Chlorophyll a-b binding protein, chloroplast precursor |
| 59 | Os09g0397700 | Glutathione-dependent formaldehyde-activating, GFA family protein |
| 60 | Os09g0439200 | Jasmonate ZIM-domain protein, Jasmonate-induced resistance to bacterial blight, Repressor of jasmonic acid signaling |
| 61 | Os09g0532000 | Senescence-inducible chloroplast protein, Activation of the chlorophll-degrading pathway during leaf senescence |
| 62 | Os10g0389200 | Red chlorophyll catabolite reductase, Leaf senescence, Wound responses |
| 63 | Os10g0415300 | Similar to Chloroplastic glutathione reductase |
| 64 | Os10g0419600 | Chlorophyllase family protein |
| 65 | Os10g0527400 | Similar to Tau class GST protein 3 |
| 66 | Os10g0527800 | Similar to Tau class GST protein 3 |
| 67 | Os10g0529500 | Similar to Glutathione-S-transferase 2 |
| 68 | Os10g0531400 | Glutathione S-transferase GST 30 |
| 69 | Os10g0575000 | JAZ-interacting transcription factor, Positive regulator of jasmonate |
| 70 | Os11g0175400 | Plant neutral invertase family protein |
| 71 | Os11g0184900 | Similar to OsNAC5 protein [imported]-rice |
| 72 | Os11g0490900 | Similar to WRKY transcription factor 72 |
| 73 | Os11g0508600 | Sugar transporter, TAL effector-mediated susceptibility to bacterial pathogen |
| 74 | Os11g0585900 | Similar to ETO1-like protein 1 |
| 75 | Os12g0277000 | Zinc finger, RING/FYVE/PHD-type domain containing protein |
| 76 | Os12g0291100 | Similar to Petunia ribulose 1,5-bisphosphate carboxylase small subunit mRNA |
| 77 | Os12g0291400 | Similar to Petunia ribulose 1,5-bisphosphate carboxylase small subunit mRNA |
| 78 | Os12g0292400 | Similar to Petunia ribulose 1,5-bisphosphate carboxylase small subunit mRNA |
| 79 | Os12g0476200 | Sucrose transporter, TAL effector PthXo2-dependent disease susceptibility to bacterial pathogen |
| 80 | Os12g0511300 | CLT |
| 81 | Os12g0512000 | Flavin monooxygenase-like enzyme , Auxin biosynthesis |
